# ‘New Year, New Me’: Restrictive Eating on Starting University

**DOI:** 10.1192/bjo.2026.11763

**Published:** 2026-06-30

**Authors:** Rokesh Arumugam, Áine McCann, Mulan Yang, Bahaa Hassan, Laurence Reed

**Affiliations:** 1 University of Cambridge, Cambridge, United Kingdom; 2 West Suffolk Hospital, Bury St Edmunds, United Kingdom

## Abstract

**Aims:**

Starting university is often the most significant challenge a young person faces - together with new freedoms, there are significant challenges. We present a case illustrating how this challenge led to the development of restricted eating, which together with delayed detection and disrupted continuity of care, led to critical malnutrition with life-threatening complications.

**Methods:**

We present the case of a 19-year-old university student who exhibited deliberate restriction of eating in her first year of university. ‘New year, new me’ - she initiated a restrictive diet and intense exercise regime of attending the gym six days a week. Rather than being weight-loss focussed, she described it as a mechanism of coping with loneliness - difficulty relating to older, final-year housemates - and environmental barriers to nutrition such as an unhygienic shared kitchen and perceptions that cooking for one was wasteful.

Parental concern at the end of her first year prompted a General Practitioner telephone consultation, followed 4 weeks later by a blood test showing hypokalaemia resulting in an Emergency Department admission. She was discharged in three days with a meal plan and a promise of a dietician follow-up call which was not fulfilled. Her parents, insistent on familial dining culture, discontinued the meal plan after one day. Subsequently, she was admitted to an acute hospital under the Medical Emergencies in Eating Disorders (MEED) pathway.

**Results:**

This case demonstrates deliberate restriction of eating emerging as an unintended consequence of attempts to regain control during a vulnerable transitional period. The move to university introduced psychosocial stressors including loneliness, disrupted routines, and environmental barriers to nutrition, aligning with literature which suggests that transitional phases increase eating-disorder risk. Service-level shortcomings, including delayed primary care assessment, fragmented follow-up, and limited continuity between acute and community services, contributed to missed opportunities for early intervention. A key paradox observed was the patient’s desire for structure and external regulation of eating illustrated through her requests for a structured meal plan, alongside significant distress when this control was imposed through nasogastric feeding. This highlights the conflicts of autonomy, control, and treatment engagement in eating disorders. Addressing these tensions requires coordinated, student-centred care pathways that integrate medical, psychological, and social support.

**Conclusion:**

Deliberate restriction of eating may develop insidiously during the transition to university, reinforced by loneliness, environmental barriers, and fragmented healthcare provision. Early recognition, and integration between primary care, acute services, and university support systems are essential to prevent progression to severe medical compromise.

